# Dispatched into disaster: a qualitative study on medical rescue teams’ personnel’s preparation, mobilization, and field living conditions after the Kahramanmaraş earthquakes

**DOI:** 10.1186/s12873-025-01416-4

**Published:** 2025-12-29

**Authors:** Ramazan Aslan

**Affiliations:** Department of Emergency and Disaster Management, Tekirdağ Namık Kemal University, Tekirdağ, Türkiye

**Keywords:** Medical rescue, Kahramanmaraş earthquakes, Preparedness, Deployment, Field logistics, Human resources

## Abstract

**Background:**

Effective disaster response requires both well-prepared medical rescue teams and resilient early-phase logistics.

**Objectives:**

This study examined how medical rescue teams’ personnel prepared, mobilized, and maintained field living conditions after the 6 February 2023 earthquakes, aiming to generate practice- and policy-oriented recommendations.

**Methods:**

A qualitative phenomenological design with purposive maximum-variation sampling (*n* = 12) was utilized. Semi-structured online interviews underwent thematic analysis. Reporting adhered to COREQ standards. Ethical approval was obtained and informed consent secured.

**Results:**

Three themes emerged. (A) *Team Structure and Personnel Characteristics*: Professional diversity enhanced capacity only when roles were explicit and leadership was visible; inconsistent training and leader ambiguity created tension. (B) *Assignment*,* Preparedness*,* and Deployment Process*: Notification and assembly often relied on informal channels (e.g., messaging apps); mission orders and destinations were unclear; winter hazards and traffic impeded deployment; initial 3–7 day rotations were perceived as most effective. (C) *Base-of-operations and logistics*: Early shelter, heating, nutrition, and hygiene support were inadequate, with privacy and sanitation being major challenges, but these improved as institutional logistics scaled up.

**Conclusions:**

The performance in the early phase can be strengthened by standardized pre-deployment checklists and mission orders, dual leadership including an experienced member, short initial rotations, and minimum living-condition standards. Competency-based modular training, regular drills, and mandatory after-action reviews are further recommended to institutionalize learning.

## Introductıon

The consecutive Mw 7.7 and 7.6 magnitude earthquakes centered in Kahramanmaraş on February 6, 2023, have been described as one of the largest disasters in the Türkiye’s history due to severe winter conditions, their impact across multiple provinces, and widespread infrastructural devastation. This disaster created a catastrophic humanitarian crisis in Türkiye [1]. United Nations situation reports in the first month indicated that millions were affected and that significant capacity gaps emerged in access to shelter and healthcare services [2].

In disasters of such magnitude, rapid access to healthcare is a fundamental component; however, infrastructural damage and a sudden surge in demand complicate the response effort. Prior capacity building and the preparedness of medical rescue teams are prerequisites for an effective response [3, 4]. Accordingly, the health response to the earthquake relied on the coordinated deployment of national capacities, including the National Medical Rescue Team (UMKE), and international Emergency Medical Teams (EMTs) [5, 6]. The role of UMKE in conducting on-site medical rescue and providing emergency health services in the disaster zone is crucial in the Turkish context [7, 8].

UMKE was established by the Ministry of Health on December 30, 2003, to provide rapid medical rescue, safe transportation, and treatment services during disasters [7]. A total of 12,749 personnel from UMKE and 112 Emergency Health Services were deployed to the earthquake zone [1]. Given the chaotic nature of disasters and the difficulty of the missions, the proper selection, comprehensive training, and adequate equipping of deployed personnel are paramount. Furthermore, the systematic organization of team formation, preparedness processes, call to duty, deployment, and on-site living conditions plays a critical role in the effectiveness and success of the services provided [9–12].

The literature presents findings that focus on different actors and contexts but often remain fragmented. The processes and challenges in medical rescue interventions for individuals with special needs have been detailed [13]. Studies on nurses who participated in the initial response to the Kahramanmaraş earthquakes reveal a focus on themes such as deployment, practical challenges, psychosocial difficulties, and positive experiences [14]. Research on first response teams highlights themes such as resources, needs, inter-agency collaboration, technological innovations, disaster management, and encountered challenges [9]. In the nursing literature, case studies from Taiwan and China demonstrate preparedness deficiencies and the need for psychological support, while also proposing experience-based theoretical frameworks [15–17]. The Kermanshah case in Iran emphasizes personal, operational, and sociocultural dimensions [12]. In Türkiye, expert opinions reveal the determinants of transportation, communication, security, coordination, and logistics; from a patient safety perspective, communication failures, resource constraints, ethical dilemmas, and psychological burden are identified as critical risks [18, 19].

Although the existing literature provides valuable findings focusing on various actors and, the vast majority of studies concentrate on the post-deployment phase. Pre-deployment are not addressed in a sufficiently systematic manner. The systematic review by Kuday et al. (2023) reveals that challenges are reported disparately across organizational, individual, environmental, health, logistical, and communication dimensions, and that a holistic, integrated framework remains absent [20]. This gap leads to limited institutional learning, particularly in the preparedness and mobilization phases, where the initial hours of a disaster are decisive.

This study aims to examine UMKE’s pre-deployment preparedness and mobilization phases, along with the sustainment of life in the field, within a single, holistic framework in the context of the February 6, 2023 Kahramanmaraş earthquakes. It seeks to generate transferable policy and practical recommendations by making decision-making, coordination, and logistical bottlenecks visible through qualitative data.

Within this scope, the study seeks to answer the following research questions:

**RQ1.** How are the structuring of UMKE teams training, and equipment standardization shaped prior to deployment?

**RQ2.** How is the notification, assembly, and deployment chain triggered and executed in the initial hours?

**RQ3.** What are the logistical and administrative constraints that determine the deployment process, and how are these constraints overcome?

**RQ4.** How are shelter, heating, nutrition, and hygiene provided for the sustainment of life in the field?

## Method

### Research design

This study is a qualitative inquiry conducted using a phenomenological design. The aim is to explore the experiences of UMKE personnel who served during the February 6, 2023 Kahramanmaraş earthquakes, focusing on the initial phase of the response. Phenomenology was selected as the most suitable approach because the research aim was specifically to understand the essence of the unique, lived experiences of personnel navigating the extraordinary circumstances of the disaster [21, 22]. This focus on the subjective meaning and essence of a shared experience makes phenomenology more appropriate than other qualitative approaches. The reporting process was structured in accordance with the COREQ checklist.

### Context and setting

The research is situated within the institutional and field realities of UMKE operations conducted in various provinces following the Kahramanmaraş earthquakes. Due to the nationwide distribution of participants, interviews were conducted online. To ensure privacy and psychological safety, sessions were scheduled outside of working hours.

### Participants, sampling, and inclusion criteria

The study employed purposive maximum variation sampling, supplemented by snowball sampling when access became difficult [22, 23]. The inclusion criteria were: (a) having actively served in the field as part of UMKE during the February 6, 2023 earthquakes, and (b) providing informed consent. Diversity in roles, seniority, and duration of service was sought. The sample size (*n* = 12) was determined by the principle of thematic saturation; recruitment was concluded when repetitions were observed that offered no new insights.

The demographic and professional characteristics of the participants are presented in Table 1. The majority of participants were in the 26–35 age range (*n* = 9) and were male (*n* = 8). In terms of professional distribution, EMTs (*n* = 5) and nurses (*n* = 4) were the most prominent groups. Most participants held a bachelor’s degree (*n* = 8). The median for professional experience was 10.5 [6.5–13.0] years, for UMKE experience was 7.0 [3.5–10.0] years, and for the total duration of service in the field was 14.5 [11.5–30] days. More than half of the participants (*n* = 7) were deployed to the field on the first day of the earthquake.

**Table 1:** Individual demographic and professional characteristics of UMKE participants (*N* = 12)

Participant code	Age group	Profession	Education level	Gender	Professional experience (years)	UMKE experience (years)	Field deployment duration (days)	Time of deployment
P01	31–35	Anesthesia technician	Bachelor’s	Male	11	10	10	Day 1
P02	26–30	Nurse	Master’s	Male	5	4	45	Day 1
P03	21–25	Nurse	High school	Male	4	3	10	Day 1
P04	31–35	EMT	Master’s	Female	13	3	15	Day 10
P05	26–30	EMT	Bachelor’s	Male	8	7	12	Day 1
P06	31–35	Anesthesia technician	Bachelor’s	Male	13	7	9	Day 9
P07	26–30	EMT	Bachelor’s	Male	8	6	14	Day 10
P08	31–35	EMT	Master’s	Male	13	11	12	Day 1
P09	26–30	Nurse	Bachelor’s	Male	13	10	30	Day 1
P10	46–50	Midwife	Bachelor’s	Female	28	15	15	Day 5
P11	26–30	EMT	Bachelor’s	Female	10	9	45	Day 1/10
P12	21–25	Nurse	Bachelor’s	Female	4	2	90	Day 1

Note. EMT = Emergency Medical Technician. Duration of service refers to the total deployment period

### Data collection tool and procedures

Data were collected through a semi-structured interview guide developed with contributions from literature and field experts. The guide covered topics such as individual and institutional preparedness; assignment and mobilization; deployment; sustainment of life in the field (shelter, heating, nutrition, hygiene); and team structure and mission duration/rotation. The guide was pre-tested for clarity and scope [24]. The key questions guiding the interviews were:

1. How would you evaluate UMKE’s current institutional structure and the structure of the team you served with?
2. Could you describe the process from the moment you received the disaster notification to your arrival at the assembly point, and finally your deployment to the field?
3. Upon arriving in the disaster zone, how did you secure your basic living conditions, and what challenges did you face?

Interviews were conducted online with the informed consent of the participants, and only audio recordings were made. All interviews were carried out by the same researcher with no third parties present. Each interview lasted between 25 and 47 min. Brief field notes were written immediately after each session. The interviews were conducted in Turkish between January and May 2024 via the Zoom platform. No repeat interviews were conducted, and no incentives or payments were provided. Interviews were transcribed verbatim following the session.

### Data analysis approach

The analysis was conducted following the reflexive thematic analysis framework by Braun and Clarke [25]. The stages included: familiarization with the data, open coding, developing candidate themes, reviewing and refining themes, and descriptive-interpretive reporting. The coding process was predominantly inductive, utilizing sensitizing concepts from logistics and organizational literature where necessary. Coding and theme management were performed using MAXQDA 2020. The analysis was carried out by a single researcher. In line with the reflexive thematic analysis approach, inter-rater reliability (IRR) was not calculated. Consistency was enhanced through a reflexive journal and peer debriefing. Turkish quotations were translated into English for reporting, and the translations were checked by a researcher to ensure no loss of meaning.

### Scientific rigor

This study adopted the trustworthiness criteria of Lincoln and Guba [26]. Credibility was established by supporting themes with direct participant quotations, presenting both representative and divergent examples, and iteratively reviewing analytical decisions. Peer debriefing was used when needed. For dependability, the data collection and analysis workflow, decision points, code definitions, and theme revisions were documented in a detailed audit trail; records in the analysis software and reflexive notes were archived. Transferability was strengthened by providing a “thick description” of the context, including the sample’s roles, seniority, service duration, and deployment locations. Confirmability was ensured by making the data-interpretation chain traceable, preserving quotation-code-theme linkages, and systematically engaging in researcher reflexivity. As member checking could only be applied to a limited extent, this is noted as a limitation of the study.

### Researcher positionality and reflexivity

The researcher has both academic and practical experience in disaster and emergency management. While this provided advantages in access and conceptual sensitivity, it also carried the risk of preconceptions. A reflexive journal was maintained throughout the process, and bracketing was applied to prevent personal experiences from directing analytical decisions. In cases of interpretive uncertainty, analytical consistency was reinforced through peer debriefing [23, 24].

### Data management and confidentiality

Audio recordings, transcripts, and analysis outputs were stored in an encrypted digital repository accessible only to the researcher. Pseudonymization was applied, and participant identities were concealed in the report using codes (P1, P2, …). Raw audio files were permanently destroyed after transcription accuracy was verified and the texts were anonymized. Data processing and storage were conducted in compliance with data protection policies. Anonymized transcripts, field notes, and analysis documents will be retained in the encrypted repository for at least five years for scientific audit and verification purposes, after which a secure destruction (irreversible deletion) procedure will be implemented.

### Ethical approval and participant rights

The study was approved by the Ardahan University Scientific Research and Publication Ethics Committee (Date: 25.12.2023, Decision No: E-67796128-000-2300041055). The research was conducted in accordance with the principles of the Declaration of Helsinki. Participants were informed about the study’s purpose, scope, voluntary nature, data usage, and potential risks, and electronic informed consent was obtained. They were reminded of their right to skip any question or withdraw from the study at any time. Sessions were paused if any discomfort was observed. The data were used for scientific purposes only and were not shared with third parties.

## Findings

The findings are reported following the three-part structure summarized in Table 2: (A) Team Structure and Personnel Characteristics, (B) Assignment, Preparedness, and Deployment Process, and (C) Base of Operations (BoO) and Logistics.

**Table 2:** Summary of the thematic framework

Main Theme	Sub-themes
A. Team Structure and Personnel Characteristics	A1. Team Structure and Composition A2. Leadership and Command System (vertical structure) A3. Team Climate and Communication (horizontal relationships) A4. Technical and Operational Competencies A5. Behavioral and Professional Competencies A6. Education, Drills, and Personnel Development A7. Motivation and Mission Engagement
B. Assignment, Preparedness, and Deployment Process	B1. Notification and Assembly B2. Individual Preparedness and Equipment Status B3. Preparation of Technical Equipment B4. Transportation and Deployment Process B5. Duration of Assignment
C. BoO and Logistics	C1. Shelter, Heating, and Sleeping Conditions C2. Nutrition and Food Supply C3. Hygiene and Sanitation C4. General Evaluations

## A. Team structure and personnel characteristics

### A1. Team structure and composition

Participants identified team composition as a critical factor, arguing that professionally heterogeneous teams were a functional advantage only if roles were clearly defined. This view was most strongly articulated by senior personnel; as one 10-year UMKE veteran (P1) stated, “*If the division of labor is handled well*,* a heterogeneous team becomes an advantage*”. This advantage, however, was contingent on relevant expertise. Participants with extensive field experience (P4, P10) warned that personnel unfamiliar with trauma care struggled and argued that “*unqualified personnel should not be deployed*”. Across experience levels, the inclusion of at least one veteran member and personnel with local knowledge was cited as critical for operational success.

Team size was also a major theme, particularly from a logistical standpoint. While large teams were seen as complicating coordination, participants with backgrounds as EMTs specifically linked effectiveness to vehicle capacity. They argued that smaller, agile units were more effective, with one (P5) noting, “Th*e coordination of teams that traveled in 5-person vehicles was easier*”. This suggests that for field-level responders, optimal team structure is both mission-specific and logistically pragmatic.

### A2. Leadership and command system (vertical structure)

A central challenge in the command system was a recurring tension between official rank and practical field experience. This critical issue was identified by participants across the experience spectrum, including both relatively junior nurses (P2, P3) and highly experienced EMTs (P8). This gap often created a command vacuum that led to internal conflicts. As one of the less-experienced participants (P3) stated, “*The lack of an official and strong team leader created conflict*”.

In contrast, participants with significant field experience were able to identify and advocate for effective solutions. For example, an 11-year UMKE veteran (P8) endorsed a dual-leadership structure, separating medical and team lead roles, as highly effective for managing complex decision-making. Beyond formal structure, a leader’s personal attitude was decisive for performance. A strong, visible leader improved morale, with one experienced EMT (P5) noting, “*A strong leader improved the team’s internal communication*”. This suggests that while junior personnel felt the impact of a command vacuum, senior personnel diagnosed its cause (rank/experience tension) and its solutions (dual leadership and strong personal competencies).

### A3. Team climate and communication (horizontal relationships)

Participants reported that the initial response phase was characterized by strong solidarity and tolerance, driven by a shared sense of purpose. As one highly experienced participant (P9) noted, “*There’s no conflict in the first 72 hours; everyone is serving a common purpose*”. During this period, internal communication was generally smooth.

However, the role of prior acquaintance revealed a significant contradiction linked to deployment timing and experience. Participants who were more experienced (P5, P6) or deployed in later, more stable phases (P4) viewed familiarity as an asset, stating, “*It’s better to work with people you know*”. In sharp contrast, a junior nurse deployed on Day 1 (P3) found that familiarity could be divisive in the initial chaotic phase, reporting that “*The prior acquaintances within the team divided it*”.

This initial cohesion was fragile and eroded as the mission extended. Conflicts arose from personnel who struggled to adapt to field conditions, the formation of internal cliques, and, as P9 also observed, periods of operational inactivity: “*As the workload decreases*,* conflict increases*”. It was noted that even a single individual could disrupt the team’s balance. In some cases, these unresolved tensions ultimately led to the departure of qualified personnel, highlighting the critical impact of team dynamics on prolonged missions.

### A4. Technical and operational competencies

Participants identified standardized knowledge and equipment proficiency as preparedness priorities. While some insisted that “Personnel must know the equipment they’ll be using very well” (P2, P4), a common challenge reported was the lack of uniform mastery over the UMKE bag, which complicated field operations.

Beyond equipment, required competencies were broadly divided into core medical skills and non-clinical operational skills. The emphasis on these skills often varied by professional role and deployment timing. For example, a senior anesthesia technician (P1) highlighted a core medical skill, stating, “*One of the fundamental skills in medical rescue is patient transport techniques*”. In contrast, the emphasis on operational skills appeared linked to the field context. Participants deployed on Day 1 (P2, P5) stressed the immediate logistical need, noting, “*Being able to set up a tent is important in UMKE*”. A participant who deployed later into a more established response (P4) expanded this requirement to larger infrastructures, such as being “*proficient with the setup… of the field hospital*”. This highlights the need for cross-disciplinary training in both medical procedures and scalable operational logistics.

### A5. Behavioral and professional competencies

Findings revealed that qualifications for UMKE personnel extend beyond technical proficiency to include crucial behavioral competencies. The perception of these competencies, however, often differed by experience level.

Seasoned participants with extensive UMKE experience (P1, P10, P11) emphasized a proactive and resilient mindset. Psychological resilience was seen as integral, with one 9-year veteran (P11) stating, “*A UMKE member should be able to manage stress very well*”. This internal resilience was linked to external action; a 10-year veteran (P1) argued that “*Being proactive in the field improves the quality of service*”, a view echoed by a 15-year veteran (P10) who stressed strong communication skills and a readiness for any assignment.

In contrast, while participants with less field experience also valued these traits, they placed a stronger emphasis on professional self-awareness and safety. One junior participant (P3) noted that a key professional attitude is “*recognizing personal limits and asking for support*”. This suggests that while senior personnel focus on maximizing their contribution (proactivity, stress management), less experienced members are focused on mitigating risk (knowing one’s limits).

### A6. Education, drills, and personnel development

Participants called for more rigorous personnel selection and development policies, emphasizing a “quality over quantity” approach. This was a clear point of consensus across the experience spectrum. The view was most strongly articulated by the sample’s most senior member, a 15-year veteran (P10), who stated, “*The quality*,* not the quantity*,* of UMKE members should be increased*”. This was echoed by one of the least experienced participants (P3), who also argued for “stricter criteria in personnel selection” to ensure a higher standard of personnel.

A major finding was that in-service training was inconsistent in both content and frequency. This criticism was particularly acute among Day 1 responders (P2, P5), who reported that “*There’s no standard in training across the teams*”. Beyond general standardization, participants identified a critical need for modular training focused on field logistics and, as noted by an experienced professional (P4), high-level “*training on command and SAKOM*”. Participants also reported that crucial learning tools like drills and After-Action Reviews (AARs) were often overlooked. One experienced member (P6) noted that his “*request for an AAR was denied*”, highlighting a systemic barrier to institutionalizing field lessons.

### A7. Motivation and mission engagement

Intrinsic motivation, driven by a desire to help and pride in the UMKE identity, was a primary factor for deployment. This was exemplified by the sample’s most senior member (P10, 15 years experience), who stated, “*I am proud to be a UMKE member*”.

However, this personal commitment was directly challenged by systemic barriers. The most significant structural barrier was the conflict with regular duty shifts, which created deployment uncertainty. “*I couldn’t go on the mission because I couldn’t find anyone to replace me*,” one participant (P6) explained. Despite such obstacles, participants displayed dedication; some (e.g., P11) deployed even after being personally affected by the earthquake.

This intense tension between high individual motivation and systemic friction fueled a central debate on the UMKE model. Notably, the strongest defense of the volunteer-based structure came from the most experienced participant (P10). In sharp contrast, a broad coalition of personnel argued for a transition to a professional, shift-based system. This view united participants regardless of deployment time or experience, from a highly experienced Day 1 responder (P9) to the sample’s least experienced member (P12). Their consensus was clear: “UMKE should be professionalized, a shift-based system like the 112 emergency service” (P6, P9, P11, P12).

## B. Assignment, preparedness, and deployment process

### B1. Notification and assembly

The initial notification and assembly process was characterized by uncoordinated communication that relied heavily on informal channels. Participants reported learning about the disaster through various means, from personal alerts to buddy systems (P02). The most common first point of contact, regardless of the eventual deployment day, was informal messaging: “*The first communication was via the WhatsApp group”.*

This lack of a standardized, formal alert system led to a chaotic assembly phase, particularly for the first wave of responders. While some teams utilized pre-determined assembly points, a Day 1 responder (P02) described the reality as “Chaos reigned at the assembly point”. This confusion stemmed from the simultaneous summoning of all personnel without clear information on time and location.

### B2. Individual preparedness and equipment status

Individual preparedness levels varied significantly, a difference strongly linked to personnel experience. Highly experienced personnel demonstrated high readiness, often keeping mission bags packed. This proactive mindset was best articulated by the sample’s most senior member (P10, 15 years experience), who deployed on Day 5: “*Because we are UMKE members*,* we are ready for a mission at any moment*”. This readiness was shared by other veterans (e.g., P1, P9) who deployed on Day 1, with some preparing for long-term self-sufficiency.

In sharp contrast was the reality for the first-responding teams. The fact that they had no time for personal preparation was widely recognized; as participants who deployed later (P04, P07) observed, “*The first team to leave had no time for individual preparation*”. This lack of time resulted in significant equipment deficiencies, such as clothing unsuitable for winter conditions. This challenge was compounded for less experienced personnel; a Day 1 responder with 3 years of experience (P3) admitted, “*I didn’t know what I needed to bring when deploying on the mission*”. Even experienced Day 1 responders (P5) reported having no time for personal packing due to urgent coordination duties, highlighting how the chaos of the initial mobilization overrode individual preparedness.

### B3. Preparation of technical equipment

Pre-deployment equipment and vehicle checks were largely routine. Most teams conducted detailed bag checks and procured necessary PPE. As one Day 1 responder (P02) noted, “*Our intervention bags were generally ready for a disaster*”. Medical consumables were typically stored separately due to expiration dates, a standard inventory management practice.

However, experiences with logistics depots varied. This variation appeared linked to both depot accessibility and internal organization. For instance, while some Day 1 participants (P03, P05) reported their depots were “very adequate” and accessible, others highlighted logistical challenges. One experienced Day 1 nurse (P09) pointed to a geographical issue: “*Our depot was far from the provincial center; it took time to bring the materials*”. Another Day 1 participant (P01) noted a systems issue: “*The locations of materials in the depots are not known by all personnel*”. This suggests that logistical planning (depot location) and internal organization (depot layout) were sources of inefficiency during the initial mobilization.

### B4. Transportation and deployment process

Teams were dispatched rapidly, often in a sequence of advance, logistics, and main units. However, this speed came at the cost of clarity, and the nature of this confusion appeared to evolve based on deployment time.

For Day 1 responders, the initial confusion was strategic and absolute. Participants deployed on the first day (P1, P2, P3) reported a complete lack of directional orders: “*We didn’t know where we were going when we deployed*.” This uncertainty shifted from strategic (where to go) to operational (what to do) upon arrival, and this second phase of confusion affected both first-wave and later-deploying teams. A broad group of participants, including those from Day 1 (P1, P2) and Day 10 (P4, P7), confirmed that their specific roles were not pre-determined: “*My assignment was determined in the province where I arrived.*”

The deployment was severely hampered by inadequate transportation planning and hazardous road conditions. These dangers were particularly acute for the first wave of responders. Participants from Day 1 teams (P5, P8, and P11) all reported experiencing “*a traffic accident due to snow*” and icy conditions while initially deploying.

### B5. Duration of assignment

A consensus emerged that initial deployments should be short, ideally 3 to 7 days for the first teams. This recommendation was most strongly articulated by highly experienced Day 1 responders (P9, P11), who had endured the initial chaos. One stated, “For the first deployed team, 5 days is ideal”. It was suggested that subsequent teams could stay longer, up to 15 days. One participant (P5) even proposed a gender-differentiated rotation based on field hardships, suggesting “5 days for women and 10 days for men”.

This ideal stood in stark contrast to the reality. While the official assignment was 10 days, actual mission durations varied significantly. Most personnel reported staying for 9 to 14 days, but some served much longer. Notably, some of the longest continuous deployments (45 to 90 days) were served by personnel with less UMKE experience (P2, P12). Redeployment was also a common practice, with many participants (e.g., P1, P2) actively requesting to return for a second mission.

## C. BoO and logistics

### C1. Shelter, heating, and sleeping conditions

In the initial days, shelter was temporary and often inadequate, a problem felt most acutely by Day 1 responders. A common experience among this first wave (P1, P2, P9, P12) was resorting to vehicles for shelter due to safety and weather concerns. Within these communal living arrangements, a lack of privacy emerged as a significant stressor. This issue was highlighted as being particularly challenging for female personnel (P5), with one (P12) noting, “There was no privacy or changing area”.

Heating was another major challenge during the initial response. A Day 1 responder (P03) confirmed this, stating, “We had a serious heating problem for the first three days”. Tent heaters consumed fuel rapidly, and supply issues left teams in severe cold. The severity of this failure was underscored by a highly experienced Day 1 responder (P11), who noted that despite extensive preparation, “Even the well-prepared personnel got cold”.

### C2. Nutrition and food supply

Nutritional conditions varied drastically, creating a sharp divide in the experiences of Day 1 responders. Some teams who deployed immediately were entirely self-sufficient; one Day 1 participant (P03) reported, “We were fully equipped with water and food”. In stark contrast, other first-responding teams faced severe hunger, with one (P02) stating, “We couldn’t eat for almost the first 30 hours”.

This initial logistical failure was overcome first through informal sharing among personnel, as local markets were non-functional. This solidarity notably transcended experience levels, uniting participants from across the seniority spectrum. Highly experienced veterans (P01, P08) and one of the least experienced members (P12) all reported the same crucial action: “We shared our food with the less-prepared personnel”. This informal support bridged the gap until institutional channels, such as logistics vehicles and the Turkish Armed Forces (TAF), became operational and stabilized the food supply.

### C3. Hygiene and sanitation

Access to toilets and showers was one of the most critical and near-unanimously cited problems. With facilities quickly overwhelmed, personnel resorted to improvised solutions. Access to showers was almost non-existent for days; as one highly experienced Day 1 responder (P08) noted, “I took my first shower on the 7th day”.

The lack of hygienic conditions negatively affected morale, and participants reported this impact was not evenly distributed, with female personnel facing greater challenges. This was observed by male colleagues, with one (P05) stating, “Women had more problems regarding the toilet”. This reality was detailed by a female participant (P12), who described having to “use a medical waste bag” or go to a “wooded area”. This failure to meet basic, gender-specific needs was seen as a significant planning oversight that lowered motivation and highlighted a severe psychological toll.

### C4. General evaluations

A broad consensus emerged among participants that UMKE teams were comparatively more prepared and operationally better equipped than other responding healthcare teams. This view was shared across the sample, uniting Day 1 responders (e.g., P3, P9), later-deployed personnel (e.g., P4, P7), and members with varying levels of experience (e.g., P3, P10, P11). One participant (P9) summarized this shared sentiment: “*UMKE teams were more prepared for the conditions compared to other healthcare workers*”.

It was also noted that logistical support improved significantly over time. Initial improvised solutions, such as the informal sharing discussed in C2, were eventually replaced by more organized institutional channels. This evolution suggests a process of institutional learning. This learning was evidenced by participants who observed post-mission improvements that directly addressed the initial gaps. Notably, it was Day 1 responders (P2, P8) and another highly experienced member (P11) who confirmed that essential supplies like “canned goods” and “toilets” were procured by the institution after the mission.

## Discussion

This study examined the field experiences of UMKE personnel following the February 6, 2023 earthquakes. The findings reveal that initial mobilization relied heavily on informal networks and improvisation, while individual preparedness levels were inconsistent, often linked to deployment timing and professional experience. The deployment phase was constrained by inadequate planning, hazardous road and climate conditions, and a lack of mission clarity, the nature of which evolved based on deployment wave. In the first 72 h, significant gaps in meeting basic needs like shelter, nutrition, and hygiene were evident, improving only as formal logistics channels became operational.

### A: Team structure and personnel characteristics

The study confirms that while professionally diverse teams offer a functional advantage, their success is contingent on strong leadership and clear roles to prevent conflict, a known challenge for volunteer teams [27, 28]. Experience was the most critical factor, as inexperienced personnel diminish performance [3, 4, 29, 30]. The absence of clear leadership created command vacuums and internal conflicts, often exacerbated by tensions between official rank and field experience [29, 31]. Although initial team solidarity was high, cohesion proved fragile over time, deteriorating due to individual adaptation issues and internal frictions [32, 33].

Effective response requires a holistic competency framework beyond standard medical skills. While baseline technical proficiency is an expected core competency [34], a lack of it reduces effectiveness [14, 35]. Our findings also confirm the need for integrated logistical and behavioral skills, such as emotional intelligence, critical thinking, resilience and proactivity [11, 15, 36]. This highlights a systemic failure: standard healthcare education is insufficient for complex emergencies, and current training is often unsystematic [29, 32, 37, 38]. The demand for standardized, continuous training is therefore critical, as trained personnel are proven to be more prepared and efficient, which boosts confidence [4, 14, 30, 33, 39].

Finally, a deep structural tension exists between the high intrinsic motivation of personnel and the sustainability of the volunteer-based model. While motivation stems from a desire to help [3], this dedication is consistently undermined by systemic barriers like duty shifts. This fuels a critical debate on professionalization. The current model’s reliance on volunteerism leads to instability and operational inefficiency, making a structural shift seem inevitable [29, 32].

### B: Assignment, preparedness, and deployment process

The mobilization and deployment phase was critically undermined by a cascade of systemic gaps, starting with an uncoordinated notification and assembly process. The heavy reliance on informal channels like WhatsApp, rather than an institutional command chain, confirms literature showing that personnel often learn of disasters through unofficial means [10, 33]. The resulting chaos at assembly points reflects a failure to maintain updated and drilled emergency plans, a systemic issue noted in the literature that leads to inefficiency in the most critical opening hours of a response [32].

This lack of institutional readiness was mirrored at the individual level. Preparedness was highly inconsistent, often depending on personal experience rather than an enforced standard, a direct link established in other studies [39]. While experienced personnel were often ready [17], the inability of first-responding teams to prepare due to time constraints contradicts the fundamental principle of 72-hour self-sufficiency [27, 40]. Issues with non-standard equipment and a lack of clear guidance underscore the necessity for institutional checklists and protocols [15, 40]. Even when teams had routine equipment checks, inaccessible or disorganized logistics depots created a significant risk, reflecting the organizational challenges that can cripple response capacity [3].

Consequently, although teams reacted quickly, the deployment itself was perhaps the weakest link. Departing without a clear mission or destination highlights a fundamental failure in managing the uncertainty of a disaster, a core challenge in the literature [41]. This was compounded by severe transportation obstacles, which directly align with studies showing that such delays reduce response effectiveness [18]. The chaotic deployment stands in stark contrast to planned models, demonstrating that individual speed is insufficient without a holistic, inter-agency transportation strategy [27]. This lack of planning also extended to mission duration, where the field-driven consensus for short, 3–7 day initial deployments aligns with established models like the Japanese DMAT system [42]. This phased approach helps contextualize the varying mission lengths found in the literature, from shorter initial deployments to longer secondary phases [15, 28, 40].

### Theme C: Boo and logistics

The findings reveal a critical logistical vulnerability in meeting the basic physiological needs of personnel, which constituted a direct operational risk. The initial failure to provide adequate shelter, heating, and sleep violated the foundational requirements for responder well-being and created a risk of “critical team loss and inefficiency” [9, 27]. This logistical vacuum, particularly the failure to meet the privacy needs of female personnel, contrasts sharply with more organized missions described in the literature [10].

This systemic gap was most evident in nutrition and hygiene. The initial period of scarcity, where some teams faced hunger, exemplifies the risks of low self-sufficiency and dependence on local resources [3, 19]. Our findings specified this risk, showing a sharp contrast in preparedness within Day 1 teams (e.g., P02 vs. P03), a gap that was initially bridged by informal peer solidarity rather than institutional support. Similarly, the lack of toilets and showers was identified as the most morale-damaging failure, confirming that hygiene is a fundamental requirement for psychological well-being, not merely a comfort factor [14, 18]. Our findings demonstrated this failure was not gender-neutral; female personnel (e.g., P12) reported greater challenges with privacy and sanitation, resorting to improvised, undignified solutions. This aligns with studies highlighting the critical need for gender-sensitive logistical planning. This gap persists in disaster response, failing to account for women’s distinct privacy and hygiene requirements, which are recognized as fundamental by humanitarian standards [43]. The fact that other major disasters have successfully managed food and hygiene logistics demonstrates that these were preventable failures, not inevitable crises [40].

Despite these severe challenges, UMKE teams exhibited a relative preparedness compared to other health groups, providing support in a chaotic environment characterized by intense resource scarcity [19]. The evolution from improvised solutions to more systematic institutional support shows a slow but functioning organizational response emerging from what the literature calls a “lack of systematic support” [3]. Most importantly, our findings contribute to the understanding of institutional learning in disaster response organizations. The post-mission steps taken, such as procuring toilets and canned goods, are promising indicators of this learning process. This specific adaptation, triggered by the field-level hardships, suggests a potential organizational shift: moving from viewing responder needs as an ad-hoc problem to recognizing responder well-being as a systemic logistical requirement that must be formally planned for [44]. This pivot from improvisation toward standardization directly addresses the critical gaps in deployment protocols and responder well-being identified throughout our findings.

### Strengths and limitations

This study is strengthened by a comprehensive, in-depth phenomenological analysis spanning disaster-response phases, maximum-variation sampling capturing diverse perspectives, and transparent thematic analysis; findings offer practical guidance for field logistics and inform institutional policy and training.

However, the findings should be interpreted within several limitations. The small sample size, while achieving thematic saturation, restricts generalizability beyond similar contexts, and data were collected only from field personnel, excluding managerial viewpoints. Online interviews may have limited non-verbal observation, and retrospective reporting could have introduced recall bias. As the analysis was carried out by a single researcher, some degree of subjectivity cannot be entirely excluded, even though reflexive measures were taken. Data triangulation was not possible, and member checking was only partially implemented. These limitations suggest that the conclusions should be read with caution and encourage future research with broader and more diverse samples to validate and extend these findings.

## Conclusion

This study examined the early response experiences of UMKE personnel following the February 6, 2023 Kahramanmaraş earthquakes. The findings suggest that during the initial response period, UMKE relied considerably on informal communication networks and individual improvisation. This situation appears to have contributed to time delays and inefficiencies in the use of resources. Responders faced major challenges in securing shelter, heating, nutrition, and sanitation during the first days of deployment, and institutional logistics became more effective only after the first seventy-two hours.

The results indicate that operational effectiveness in disaster medical response is influenced by structural and procedural conditions. Professional diversity among team members can support performance when roles are clearly defined and leadership is visible. In contrast, unclear authority and limited experience may lead to coordination problems. Similarly, the observed mobilization difficulties show that standardized pre-deployment procedures, including clear mission orders and readiness checklists, could improve coordination and speed. The gaps in living conditions also highlight the importance of establishing minimum responder well-being standards as part of formal logistics planning rather than leaving them to improvisation.

Overall, the study points to the need for more systematic organization in deployment and logistics processes. However, the findings should be interpreted within the limitations of a qualitative design with a small sample size. Although the results cannot be generalized to all response organizations, they provide useful insights into the organizational learning needs of medical rescue teams in large-scale disasters.

### Recommendations

Based on the findings, several practical suggestions can be made to strengthen UMKE’s operational capacity and the well-being of its members. Pre-deployment and mobilization processes could be improved through a unified digital alert and communication system supported by standardized mission orders and readiness checklists. Regular modular training programs that emphasize both professional and logistical skills would help ensure a more consistent level of preparedness across regions. Leadership can be enhanced by assigning two complementary leaders who share medical and operational responsibilities and by ensuring that each team includes at least one experienced member. Institutional policies should also guarantee basic living standards in the field, such as adequate shelter, heating, nutrition, hygiene, and privacy, while considering gender-sensitive needs. In addition, after-action reviews and structured data collection should become a routine part of operations to strengthen institutional memory and continuous improvement. Future research may examine these recommendations through broader samples and different contexts to test their general applicability and effectiveness.

## Data Availability

The datasets generated and/or analyzed during the current study are not publicly available due to privacy restrictions but are available from the corresponding author on reasonable request.

## Declarations

### Translation assistance

The English translation was assisted by ChatGPT and subsequently reviewed and verified by the author.
